# Planarians Sense Simulated Microgravity and Hypergravity

**DOI:** 10.1155/2014/679672

**Published:** 2014-09-17

**Authors:** Teresa Adell, Emili Saló, Jack J. W. A. van Loon, Gennaro Auletta

**Affiliations:** ^1^Department of Genetics and Institute of Biomedicine, University of Barcelona, Catalonia, 08028 Barcelona, Spain; ^2^Dutch Experiment Support Center (DESC), Department of Oral and Maxillofacial Surgery/Oral Pathology, VU University Medical Center & Academic Centre for Dentistry Amsterdam (ACTA), Gustav Mahlerlaan 3004, 1081 LA Amsterdam, The Netherlands; ^3^ESA-ESTEC, TEC-MMG, Keplerlaan 1, 2200 AG Noordwijk, The Netherlands; ^4^Pontifical Gregorian University, Piazza della Pilotta 4, 00187 Roma, Italy; ^5^University of Cassino, Via Zamosch 43, 03043 Cassino, Italy

## Abstract

Planarians are flatworms, which belong to the phylum *Platyhelminthes.* They have been a classical subject of study due to their amazing regenerative ability, which relies on the existence of adult totipotent stem cells. Nowadays they are an emerging model system in the field of developmental, regenerative, and stem cell biology. In this study we analyze the effect of a simulated microgravity and a hypergravity environment during the process of planarian regeneration and embryogenesis. We demonstrate that simulated microgravity by means of the random positioning machine (RPM) set at a speed of 60 °/s but not at 10 °/s produces the dead of planarians. Under hypergravity of 3 g and 4 g in a large diameter centrifuge (LDC) planarians can regenerate missing tissues, although a decrease in the proliferation rate is observed. Under 8 g hypergravity small planarian fragments are not able to regenerate. Moreover, we found an effect of gravity alterations in the rate of planarian scission, which is its asexual mode of reproduction. No apparent effects of altered gravity were found during the embryonic development.

## 1. Introduction

Planarians, commonly known as flatworms, are bilaterally symmetric metazoans of the phylum* Platyhelminthes*. They are unsegmented and acoelomate and possess well-defined anteroposterior and dorsoventral axes. They show an anterior cephalic region containing the brain and a pair of eyespots, a central region with the pharynx and the mouth opening, and a posterior tail region. Planarians ability to regenerate complete animals from any fragment of their body has attracted the interest of scientists long ago. However, it has been in the last years that planarians have become an essential model in the field of regeneration and stem cells [1–4]. The reason is the development of genomic tools to analyse its regeneration process from a molecular perspective and the demonstration that the feature underlying its regeneration potential is the existence of a unique population of adult totipotent stem cells, known as neoblasts [5, 6]. Planarians possess complex organs as the pharynx and the central nervous system (CNS), which conserves all neural cell types and neuropeptides found in vertebrates [7]. Thus the study of the mechanisms that enable planarians to regenerate such structures will give insight into the genetic and molecular paths that enable such capacity. In the last years the essential role of the continuous activation of the intercellular signalling pathways, such as the Wnt or the BMP, to enable proper patterning during regeneration has been extensively demonstrated [8, 9].

Earth gravity is a constant parameter in living organisms which has had to be an essential environmental parameter along evolution. Accordingly, microgravity has been shown to alter the structure of cells, as well as their apoptotic and mitotic responses [10–12]. However, the cellular effects of microgravity are poorly understood. Our project focuses on the study of the regenerative capacity of planarians under micro- and hypergravity conditions with respect to normal earth gravity environment [13]. Alteration of the gravity conditions impacts on the establishment of cell polarity, intercellular communication and the expression of related genes, which are essential mechanisms involved in cell proliferation and differentiation, and thus in regeneration. Therefore, gravity should be a relevant parameter during planarians regeneration.

Using the planarian species,* Schmidtea mediterranea*, which reproduces strictly asexually, we demonstrate that simulated microgravity and hypergravity changes have an impact during the process of planarian regeneration. We found that different settings in the random positioning machine (RPM) [14, 15] to simulate microgravity produce different effects on planarians. Using a second planarian species,* Schmidtea polychroa*, which shows both asexual and sexual modes of reproduction, no apparent effects of altered gravity were found during the embryonic development.

## 2. Materials and Methods

### 2.1. Planarian Culture

Asexual planarians used in the regeneration experiments were from a clonal strain of the* Schmidtea mediterranea* BCN-10 biotype and were maintained as previously described [16]. Planarians were 4 to 6 mm in length when used for experiments. A sexual population of* Schmidtea polychroa* was used in the embryological studies. It was collected in the Tordera river from Sant Celoni (Barcelona, Spain) and was maintained in the lab as described elsewhere [17]. Three to six day old cocoons were used for the experiment.

### 2.2. Experimental Procedure

Intact asexual planarians and cocoons were placed in 50 mL falcon tubes for their transport from Barcelona to ESTEC (European Space Research and Technology Centre, Noordwijk, The Netherlands), where simulation facilities are located. At day 0 planarians or cocoons were distributed in falcons, at a density of 10 animals or cocoons per falcon. Each falcon was filled with planarian water taken from Barcelona. Animals were kept in the dark and at a constant temperature of 21°C. The results presented correspond to 3 independent experiments. Experiment 1 corresponds to the analysis of* S. mediterranea* trunk fragments regeneration in RPM (Dutch Space, Leiden, The Netherlands) or LDC 3 g during 13 days. The RPM was set to real random mode and random direction with a maximum speed of 60°/s. The samples were fixed in the centre of the inner frame with a resulting largest radius of 9 cm to the outermost sample resulting in a maximum residual g due to rotation of less than 10^−4 ^g [14]. RMP controls were placed in the same environment as the RPM. The LDC controls were placed at the center of the centrifuge in order to expose the samples to the same angular motion but without the additional gravitational load. Animals were loaded intact at day 0 (d0). At d 1 they were taken out of the falcon and cut in head, trunk, and tail pieces. Trunk pieces were loaded again in the previous environment. All samples were analysed at d 13. Forty planarians distributed in 4 falcons were analysed per condition. Experiment 2 corresponds to the analysis of* S. mediterranea* regeneration in RPM 60°/s and 10°/s and in LDC 4 g and 8 g. In this experiment four experimental groups were analysed: Group 1 was composed of trunk planarian fragments cut at d 1 of the experiment and analyzed at d 6. Group 2 was composed of trunk planarian fragments cut at d 1 of the experiment and analyzed at d 13. Group 3 was composed of trunk planarian fragments cut at d 0 of the experiment and analyzed at d 13. Group 4 was only included in the LDC and was composed of head, trunk, and tail fragments cut at d 0 and analyzed at d 13. Forty planarians distributed in 4 falcons were analysed per condition. Experiment 3 (which chronologically was the first) corresponds to the analysis of planarian embryogenesis using* S. polychroa* 3–6d old cocoons in RPM 60°/s or LDC 3 g conditions during 13 days. Ten cocoons per condition were analysed. In all experiments, at the corresponding day (d 6 or d 13) animals were taken out, analysed by morphological inspection at naked eye and at the stereomicroscope. Afterwards animals were fixed in Carnoy for the subsequent immunological staining as described [18]. A corresponding control group at 1 g was included for every RPM and LDC experimental group.

### 2.3. Immunostaining

Immunostaining was carried out as described previously [18]. The following antibodies were used: anti-synapsin (anti-SYNORF1, 1 : 50; Developmental Studies Hybridoma Bank), anti-arrestin (VC1, a kind gift of Professor H. Orri), anti-phosphohistone H3 (Ser10) (D2C8) (pH3) (1 : 500; Cell Signaling Technology), and anti-α-tubulin (1 : 20, Developmental Studies Hybridoma Bank). Confocal laser scanning microscopy was performed using a Leica TCS 4D (Leica Lasertechnik, Heidelberg) adapted for an inverted microscope (Leitz DMIRB). Images were processed using ImageJ software. p-H3 positive cells were counted by visual inspection of confocal z-stacks of images corresponding to the whole animal. In the corresponding graphs error bars represent standard error of the mean.

## 3. Results and Discussion

### 3.1. Planarians Can Regenerate in 3 g Hypergravity but Not in the Microgravity Simulated by the 60°/s Random Positioning Machine

**Figure 1:**
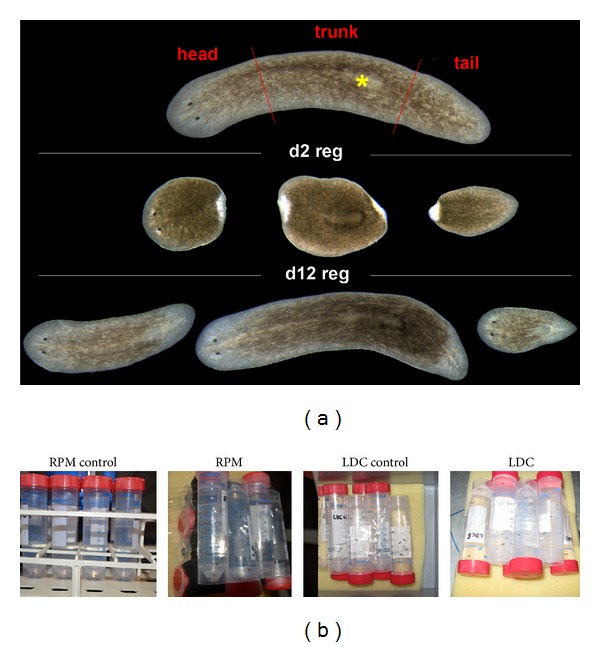
(a) Planarians' regeneration timing. When planarians are cut into head, trunk, and tail pieces, they produce a blastema (unpigmented region at day 2), and the subsequent regeneration of all missing tissues in 10–15 days. The yellow asterisk labels the pharynx. (b) The two environments in which planarians were cultured both with sample and control groups. Animals were included in falcon tubes, filled with planarian water, at a density of 10 animals per falcon.

**Figure 2:**
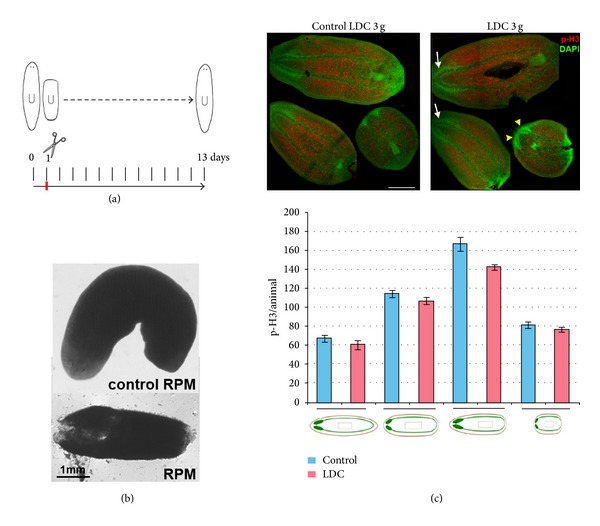
Results of Experiment 1. Planarians regenerate in 3 g hypergravity but not in the microgravity simulated by the 60°/s RPM. (a) Schedule of Experiment 1. (b) Comparison between sample group and control group in the RPM. At day 13 all RPM animals appeared dead, while controls were regenerated and healthy. (c) Comparison between sample group and control group in the LDC. DAPI staining (green) shows that both groups regenerated properly the missing structures. In both groups animals fissioned during the experiment and thus at day 13 animals at different stages of regeneration are found. In the image, a completely regenerated brain in the anterior region is labelled with white arrows and a regenerating one of a planarian fragment is indicated by a yellow arrowhead. The counting of pH3 positive cells in LDC planarians, grouped according to their morphology after their differential fissioning, indicates that under hypergravity conditions there is a slight decrease in the number of proliferative cells, although it is not statistically significant. Graphs error bars represent standard error of the mean. Data were analyzed by Student's *t*-test. Differences are considered significant at *P* < 0.05.

To assess the effect of simulated altered gravity during planarians regeneration, in a first experiment (Experiment 1 in material and methods) planarians were cut in three pieces (head, trunk, and tail), and only trunk fragments were cultured in RPM or LDC simulators [14, 15, 19]. Although all three pieces are able to regenerate complete planarians in few days (Figure 1(a)), only trunk pieces, which must regenerate the head and tail regions, were assayed in this experiment. At day 0, intact animals were distributed in 50 mL falcons (10 planarians/falcon) and set in the corresponding condition (Figure 1(b)). Two conditions were studied: simulated microgravity by a random positioning machine (RPM) and hypergravity (3 g) by a long diameter centrifuge (LDC) (Figure 1(b)). For each condition a control group was included. Since we reasoned that a pretreatment of the animals with simulated altered gravity could produce stronger effects during the earliest regeneration stages, at day 0 planarians were not cut but were laid intact in the falcons (Figure 2(a)). At day 1, planarians were taken out of the falcons and cut. The corresponding trunk pieces were again included in the falcons and returned to the corresponding environment. Head and tail pieces were discarded. After 12 days in the corresponding conditions (day 13 of the experiment) planarians were analyzed. Unexpectedly, all animals that had been in the RPM machine were dead, while their corresponding controls appeared healthy and completely regenerated (Figure 2(b)). Taking into account that dead planarians disaggregate in the medium in few hours and that extracted RPM sample planarians still conserved a recognizable structure (see Figure 2(b)), we conjectured that simulated microgravity did not cause the immediate death of planarians but they rather died close before the end of the simulation.

LDC planarians at 3 g appeared completely regenerated and undistinguishable of their respective controls. A structural analysis using DAPI, which labels the cell nucleus, showed no differences within 3 g animals and their controls. Both showed regenerated brain and tail tissues (Figure 2(c)). The only remarkable observation is that, in both groups, animals suffered spontaneous scission of the tail. Asexual reproduction is the only way of reproduction of the planarian species used in this study,* Schmidtea mediterranea*. This mode of reproduction consists in the narrowing of the postpharyngeal region and the subsequent scission of the tail, which eventually regenerates a complete planarian in few days. The original animal regenerates the tail also in few days. The signals that induce planarians to fission are not clearly understood, but changes in the medium, the temperature, or the density of individuals in the environment are all factors that directly influence the process. For that reason, the finding of spontaneous scission in our animals was not surprising. Note that animals fissioned across the whole duration of the experiment, since we found planarians that missed the tail but showed perfectly regenerated cephalic ganglia as well as animals that were just starting to regenerate the new cephalic ganglia (see Figure 2(c)). To further assess a possible effect of hypergravity in planarians, we analysed the rate of proliferation by using an antibody against phosphorilated-H3 (Ser10) (pH3), which labels cells in M phase (Figure 2(c)). Since at the end of the experiment planarians were not at the same stage of regeneration because of the scission that some animals suffered and because mitotic rates directly depend on the regeneration stage, animals were grouped according to their regeneration stage, inferred from the DAPI staining, to quantify pH3 positive cells. The quantification showed slight differences between LDC 3 g and control animals, with mitotic rates being lower in LDC 3 g planarians of all groups (Figure 2(c)).

The results of this experiment demonstrate that the microgravity simulated by the RPM machine in the present experimental conditions is lethal for planarians. However, it does not seem to be lethal from the very beginning but after several days of treatment. Moreover, our results demonstrate that, under 3 g hypergravity, although physiological parameters as proliferative rates could be affected, planarians can regenerate perfectly the missing structures.

### 3.2. RPM at 10°/s Is Not Lethal for Planarians

**Figure 3:**
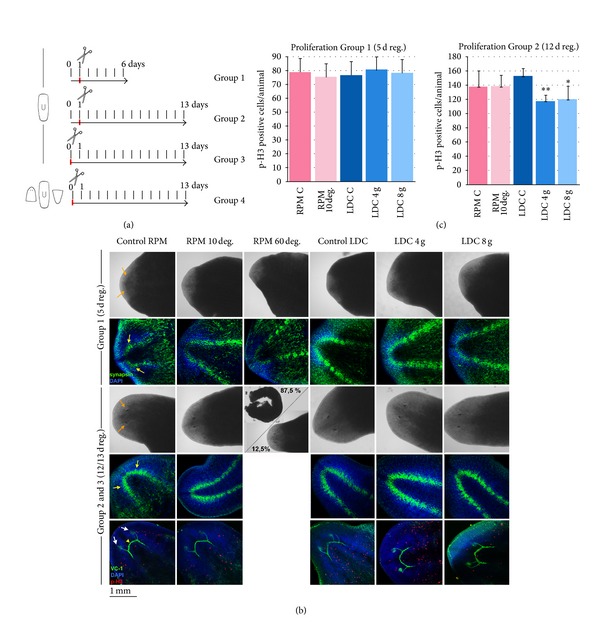
Results of Experiment 2. Planarian trunks can regenerate properly in the RPM at 10°/s and under 4 g and 8 g LDC hypergravity. (a) Schedule of Experiment 2. (b) At day 5 all animals are regenerating properly (see the two black spots in the in vivo image and the brain stained with DAPI, in blue, and anti-synapsin, in green). At day 13 all animals are properly regenerated but RPM 60°/s, which were dead in 87,5% (see the eyes, the brain stained with anti-synapsin and the visual axons stained with anti-VC1). (c) The counting of pH3 positive cells in RPM and LDC planarians at days 5 and 12 of regeneration indicates that under hypergravity there is a significant decrease in the number of proliferative cells at 12 days of regeneration. Graphs error bars represent standard error of the mean. Data were analyzed by Student's *t*-test. Differences are considered significant at *P* < 0.05. Orange arrows indicate the regenerating eyes. Yellow arrows indicate the regenerating brain. White arrows indicate the regenerating eyes stained with anti-VC1. Yellow arrowhead points to the optic chiasm.

In order to test whether the lethal effects produced in planarians in RPM Experiment 1 were due to the settings for simulated low gravity or to the specific rotation movement induced by the RPM machine, we performed Experiment 2, in which a part of the samples was cultured in a second RPM set to rotate at a maximum rotation speed of 10°/s. A group of planarians was also cultured in the previous conditions (max speed 60°/s), in order to reproduce the lethal effects. In the present experiment planarians were also included in the LDC, in order to test the effect of higher gravity (4 and 8 g) (see next section). Three groups of planarians were analysed in the simulated microgravity experiment (Figure 3(a)). Group 1 and Group 2 comprised planarians in which the cut of the head and tail was performed one day after their incubation in the RPM machines, like in the previous experiment. Group 1 planarians were analysed at day 6 of the experiment (day 5 of regeneration), and Group 2 planarians were analysed at day 13 of the experiment (day 12 of regeneration), like in the previous experiment. Group 3 planarians were also analysed at day 13, but they were cut before any pretreatment, at day 0. The reason for including this group was the possibility that the preincubation in simulated microgravity could have stronger effects in planarians. Our results show that all Group 1 planarians appeared properly regenerated like controls, showing eyes (see two black spots in Figure 3(b)) and a CNS (see anti-synapsin and DAPI labeling in Figure 3(b)). However, in Groups 2 and 3, planarians loaded into the 60°/s RPM machine were almost all dead, whereas planarians loaded into the 10°/s RPM machine and controls appeared perfectly regenerated (see in vivo and immunostained images in Figure 3(b), showing the eyes and the CNS). Further analysis of the mitotic activity in 10°/s RPM animals and their controls showed no differences neither at 5 nor at 12 days of regeneration.

These results demonstrate that it is not the simulated microgravity as such but the specific setting of rotation of the 60°/s RPM machine which produces the death of planarians. Moreover, the finding that all planarians appeared healthy and properly regenerated according to the regeneration stage in Group 1 (day 5) represents evidence for the fact that those planarians do not die soon after sensing the effects of the 60°/s RPM machine but later on, that is, after having regenerated the main structures. One explanation could be that the specific rotation produced by the 60°/s RPM machine but not by the 10°/s RPM affects specific sensory organs that planarians possess around the head, the rheoreceptors, which sense the water currents [20]. The inclusion of a control group without suffering changes of direction but only fluid motion would help to clarify this issue. The possibility that the sensitivity of the rheoreceptors is the cause of planarians dead in the 60°/s RPM machine would explain that planarians only die after having regenerated the head, where rheoreceptors are found. It would be necessary to analyse the effect of 60°/s RPM rotation in intact animals to definitely corroborate this hypothesis. Intermediate speeds of the RPM can be used to identify the sensitivity threshold of this effect.

### 3.3. Planarian Trunks but Not Head and Tail Fragments Can Regenerate Properly under 4 g and 8 g Hypergravity

Since we knew from Experiment 1 that 3 g hypergravity did not produce any apparent effect on planarians regeneration, in Experiment 2 we tested whether rising hypergravity up to 4 and 8 g would allow planarians to properly regenerate the missing structures. At both days 6 and 13 of the experiment all LDC planarians showed regenerated head structures undistinguishable from the controls (see eyes and CNS in Figure 3(b)). Quantification of mitotic rates of 4 and 8 g LDC planarians showed that, although at 5 days of regeneration there are no differences with respect to the controls, at 12 days both 4 and 8 g LDC planarians show a significant decrease.

**Figure 4:**
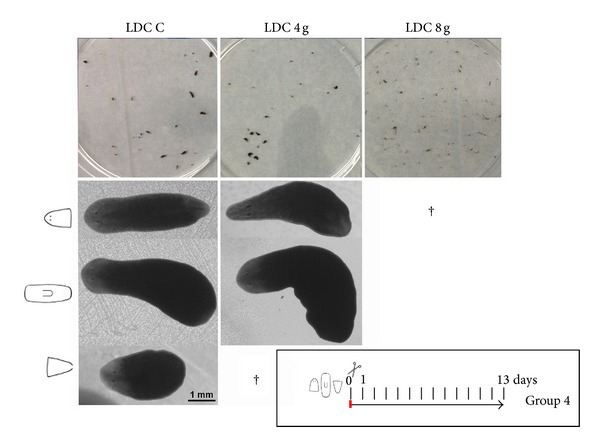
Hypergravity impairs regeneration of small planarian fragments. General images of the different planarian groups in the LDC conditions at the end of Experiment 2 are shown in the upper panel. Note that at 8 g all planarians are dead. In the lower panel stereoscopic images of planarians show that, while control fragments regenerated properly, head fragments under 4 g conditions present an aberrant morphology. Moreover, no tail fragments were found, which means they died during the experiment.

A fourth group of planarians (Group 4) was analyzed in hypergravity conditions, in which not only trunk but head and tail pieces were also included. The aim was to test the possibility whether, although trunk fragments regenerate properly under hypergravity, smaller fragments which are forced to regenerate big parts would be more sensitive to gravity changes. We found that while all control fragments could regenerate the missing structures, all planarians of Group 4 under 8 g conditions were dead at the end of the experiment (Figure 4). Regarding 4 g planarians, trunk fragments appeared healthy and regenerated. However, head fragments of 4 g planarians were regenerated but unhealthy since the head was thinner and the animals showed aberrant motility behaviour (Figure 4). Moreover, we could not find tail fragments, which means that they did not survive. The fact that trunk fragments at 8 g of Groups 2 and 3 do survive and regenerated properly seems to be contradictory with the finding that all fragments of Group 4 died at 8 g, since we would expect that at least trunk fragments also survive. However, it is known that massive death of planarians in a specific container produce also the death of their neighbours, even if they are healthy. The reason could be a highest sensitivity to infections and/or the release of toxic solutes from the death tissues.

Altogether these data demonstrate that a high increase in the gravitational force (4–8 g) during planarians regeneration does affect the capability to regenerate and that smaller fragments are more sensitive to this effect. Although the molecular reason of this effect is not known, the finding that proliferative rates are decreased would suggest that, as it happens in other organisms, gravity could affect the actin cytoskeleton and the assembly of microtubules, from among other subcellular parameters [21–23].

### 3.4. Gravity Could Affect Planarian Asexual Reproduction

**Figure 5:**
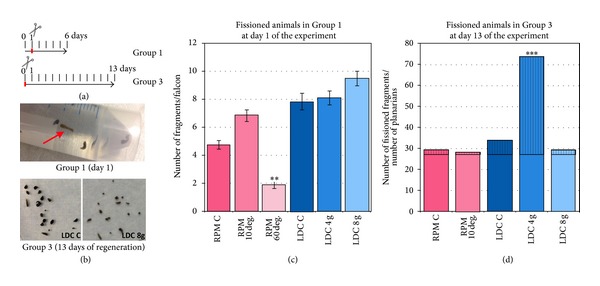
Simulated alteration of gravity affects planarian scission rate. (a) Scheme of the groups corresponding to Experiment 2 in which scission rates were analyzed. (b) At day 1 several fissioning animals were found (red arrow, in upper images). At day 13 a higher number of small fragments were found in LDC 8 g conditions compared to controls (lower images). (c) Quantification of the fissioned animals at day 1 of the experiment shows that RPM (60°/s) decreases while LDC (8 g) increases it. (d) Quantification of the number of small fissioned fragments in relation to the number of original planarians shows a higher rate of fissioned animals under 4 g hypergravity. In the graphs error bars represent standard error of the mean. Data were analyzed by Student's *t*-test. **P < 0.001; ***P < 0.001. Differences are considered significant at *P* < 0.05.

Since in Experiment 1 we observed a high rate of scission in planarians, we decided to analyse and quantify it accurately in Experiment 2. We quantified the number of planarian fragments at two time points: at day 1, when Groups 1 and 2 were removed from the falcons and cut; and at the end of the experiment (day 13). At day 1 we could observe several planarians that were stretched and attached to the falcon wall, which is the typical sign of scissioning (see Figure 5(b)). When counting the fragments in each group, we found that 10°/s RPM planarians had suffered a significant increase in the fissioning rate compared to controls and 60°/s RPM planarians. On the contrary, LDC 8 g planarians showed a slightly higher scission rate when compared to their own controls (Figure 5(c)).

In Group 3 planarians, at day 13, we quantified the big planarian fragments, which were assumed to be the original trunks, and the smaller ones, which were assumed to be generated by the scission. Interestingly, we found that the number of smaller fragments was significantly higher in LDC 4 g planarians compared with the rest of groups (Figure 5(d)).

These results together indicate that simulated microgravity could inhibit fissioning, that is, the asexual mode of planarian reproduction, while hypergravity would induce it. The fact that we did not find more fissioned fragments in 8 g LDC planarians could mean that above a threshold of the gravity force there is no increase of the fissioning rate. The effect of gravity on the fissioning rate could be also related to its role in maintaining the structure of the cell cytoskeleton and its influence in cell shape and tissue density or hardness.

### 3.5. Planarian Embryos Develop Normally under Simulated Microgravity and under Hypergravity

**Figure 6:**
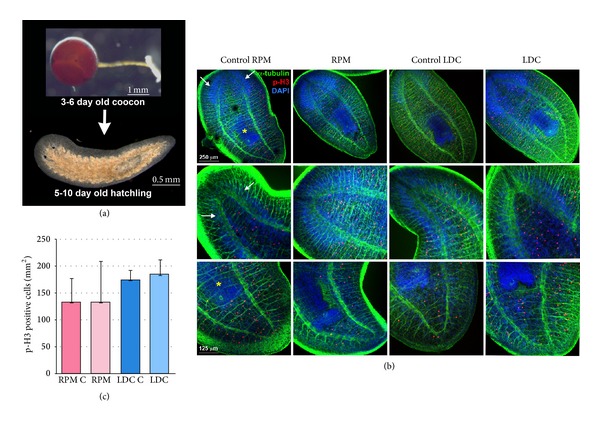
Planarian embryos develop in simulated microgravity (RPM 60°/s) and in hypergravity (3 g) environments. (a) Image of a planarian cocoon and a hatchling planarian. (b) Immunohistochemical analysis of planarian hatchlings corresponding to all conditions tested shows their normal morphology (see the nervous system stained with anti-α-tubulin, in green, and the pharynx and brain stained with DAPI, in blue). White arrows indicate the brain and yellow asterisk indicates the pharynx. (c) Quantification of the pH3 shows no significant differences in the mitotic rates among the different groups. Data were analyzed by Student's *t*-test. Differences are considered significant at *P* < 0.05.

To analyse if micro- or hypergravity could have an impact on planarians embryonic development we performed Experiment 3 (see Materials and Methods) using the planarian species* Schmidtea polychroa*, which reproduces both sexually and asexually.* S. polychroa* lays polyembryonic eggs, named cocoons, which contain several embryos within a mass of yolk cells. Fifteen days after deposition juveniles hatch from the cocoons, which already show the morphology and molecular features of adult planarians [24] (Figure 6(a)). Three to six day old cocoons of* S. polychroa* were cultured in simulated microgravity conditions in the 60°/s RPM or in hypergravity conditions, at 3 g, in the LDC. Thirteen days later, the new hatchlings were fixed and analysed by “whole mount” immunohistochemistry. In all conditions juveniles showed a proper morphology and organization of the CNS and pharynx, as showed with the anti-α-tubulin antibody and DAPI stainings (Figure 6(b)). Through immunohistochemistry using the anti-pH3 antibody the mitotic activity was quantified, demonstrating no significant differences among the groups (Figure 6(c)).

Those results demonstrate that planarians can develop properly in simulated microgravity and under hypergravity conditions, at least in the specific settings used in the present experiment. The fact that hatchlings did not die in the RPM 60°/s conditions, as observed with adult planarians, could be explained by the fact that during most of the experiment those animals were still developing the structures inside the cocoon, like the rheoreceptors. We predict that the permanence of some more days in the RPM would also induce the death of those newborn animals.

## 4. Conclusions

About regeneration in microgravity conditions, planarians experience lethal conditions with the RPM set at maximum speed of 60°/s but not with the RPM set at 10°/s. Our conclusion is that it is not the simulated microgravity as such but the specific setting of rotation of the 60°/s RPM machine which produces the death of planarians. Moreover, in the latter case, planarians do not die soon after sensing the effects of the 60°/s RPM machine but after having regenerated the main structures. We have hypothesized that planarians die after having regenerated the head, since the rotation of the RPM 60°/s affects specific sensory organs that planarians possess around the head, the rheoreceptors, which sense the water currents. Further analysis is needed in order to definitely corroborate this hypothesis. Planarians regenerated in the RPM 10°/s environment do not show any apparent defect in the new structures or in their proliferative rates. A transcriptomic analysis of those samples is under study, which will clarify whether although simulated microgravity does not interfere in the ability to regenerate it produces changes at the genetic level.

About regeneration in hypergravity conditions, we have found that 3 g does not affect the regeneration rate, while a high increase in the gravity force (up to 4 g and 8 g) during planarians regeneration does affect the capability to regenerate, and that smaller fragments are more sensitive to this effect. In particular, they are still able to regenerate from trunk fragments but not from tails and heads. This effect, together with the finding that planarians in the LDC environment show a decrease in the proliferation rates, suggests that, as it happens for other organisms, gravity could affect the actin cytoskeleton and the assembly of microtubules, from among other subcellular parameters.

Moreover, our results indicate that microgravity could inhibit fissioning, that is, the asexual mode of planarian reproduction, while hypergravity would induce it. It is also likely that above a certain gravity threshold there is no increase of the fissioning rate. This observation agrees with the possible impact of gravity force in the assembly of microtubules and the organization of the cell cytoskeleton.

Finally, in all RPM 60°/s and LDC 3 g conditions juveniles could develop from the cocoons and showed a proper morphology and organization of the CNS and pharynx. No significant differences in the rates of proliferation were found among the groups.

All these results together show that planarians are a suitable organism to be tested in both microgravity and hypergravity environments. Moreover, the fact that these environments affect both fission and regeneration rates makes these organisms interesting for testing effects that could provide us with important insights about basic molecular processes occurring in a wide range of vertebrates, including humans.

## References

[1] Saló E, Baguñà J (2002). Regeneration in planarians and other worms: new findings, new tools and new perspectives. *Journal of Experimental Zoology*.

[2] Reddien PW, Alvarado S (2004). Fundamentals of planarian regeneration. *Annual Review of Cell and Developmental Biology*.

[3] Saló E (2006). The power of regeneration and the stem-cell kingdom: freshwater planarians (Platyhelminthes). *BioEssays*.

[4] Alvarado AS (2006). Planarian regeneration: its end is its beginning. *Cell*.

[5] Handberg-Thorsager M, Fernandez E, Saló E (2008). Stem cells and regeneration in planarians. *Frontiers in Bioscience*.

[6] Baguñà J (2012). The planarian neoblast: the rambling history of its origin and some current black boxes. *International Journal of Developmental Biology*.

[7] Fraguas S, Barberán S, Ibarra B, Stöger L, Cebrià F (2012). Regeneration of neuronal cell types in Schmidtea mediterranea : an immunohistochemical and expression study. *The International Journal of Developmental Biology*.

[8] Almuedo-Castillo M, Sureda-Gómez M, Adell T (2012). Wnt signaling in planarians: new answers to old questions. *International Journal of Developmental Biology*.

[9] Molina MD, Saló E, Cebrià F (2011). Organizing the DV axis during planarian regeneration. *Communicative & Integrative Biology*.

[10] Sarkar D, Nagaya T, Koga K, Seo H (1999). Culture in vector-averaged gravity environment in a clinostat results in detachment of osteoblastic ROS 17/2.8 cells. *Environmental Medicine*.

[11] Uva BM, Masini MA, Sturla M (2002). Clinorotation-induced weightlessness influences the cytoskeleton of glial cells in culture. *Brain Research*.

[12] Sundaresan A, Risin D, Pellis NR (2002). Loss of signal transduction and inhibition of lymphocyte locomotion in a ground-based model of microgravity. *In Vitro Cellular & Developmental Biology—Animal*.

[13] Auletta G, Adell T, Colagè I, D'Ambrosio P, Salò E (2012). Space research program on planarian *Schmidtea mediterranea*'s establishment of the anterior-posterior axis in altered gravity conditions. *Microgravity Science and Technology*.

[14] van Loon JJWA (2007). Some history and use of the random positioning machine, RPM, in gravity related research. *Advances in Space Research*.

[15] Borst AG, van Loon JJWA (2009). Technology and developments for the random positioning machine, RPM. *Microgravity Science and Technology*.

[16] Fernandéz-Taboada E, Moritz S, Zeuschner D (2010). Smed-SmB , a member of the LSm protein superfamily, is essential for chromatoid body organization and planarian stem cell proliferation. *Development*.

[17] Martín-Durán JM, Duocastella M, Serra P, Romero R (2008). New method to deliver exogenous material into developing planarian embryos. *Journal of Experimental Zoology B*.

[18] Alvarado AS, Newmark PA (1999). Double-stranded RNA specifically disrupts gene expression during planarian regeneration. *Proceedings of the National Academy of Sciences of the United States of America*.

[19] van Loon JJWA, Krause J, Cunha H, Goncalves J, Almeida H, Schiller P The large diameter centrifuge, LDC, for life and physical sciences and technology.

[20] Skaer RJ (1961). Some aspects of the cytology of *polycelis nigra*. *Quarterly Journal of Microscopical Science*.

[21] Papaseit C, Pochon N, Tabony J (2000). Microtubule self-organization is gravity-dependent. *Proceedings of the National Academy of Sciences of the United States of America*.

[22] Crawford-Young SJ (2006). Effects of microgravity on cell cytoskeleton and embryogenesis. *International Journal of Developmental Biology*.

[23] Vorselen D, Roos WH, MacKintosh FC, Wuite GJ, van Loon JJ (2014). The role of the cytoskeleton in sensing changes in gravity by nonspecialized cells. *The FASEB Journal*.

[24] Martín-Durán JM, Monjo F, Romero R (2012). Planarian embryology in the era of comparative developmental biology. *The International Journal of Developmental Biology*.

